# Split difference method to determine the polynomial function that models the first few given terms of a sequence

**DOI:** 10.1016/j.mex.2020.100956

**Published:** 2020-06-19

**Authors:** Rithvik Ravikumar

**Keywords:** Quadratic functions, Polynomials, Sequences, Split, Difference

## Abstract

When looking at a sequence of numbers, one that can be defined by a polynomial function of a natural number degree, one most commonly would use a difference table to find the degree followed by a system of equations to find the equation that models the sequence. This method can prove to be very time consuming as solving a system of equations can become tedious at higher degrees. Alternately, some people would use a method where they use the difference table to find the leading coefficient in addition to the degree to give the first term. Then they would subtract this term from the function and repeat this process. However, this can be unnecessarily complicated as this method requires one to create a difference table numerous times only to need the last difference. This method uses a simple pattern triangle and only the first difference table of the sequence. It is already necessary to create the first difference table and this pattern triangle can be used to improve upon the second method. The pattern triangle allows us to walk through the difference table of the lower degree polynomials quite easily, removing the need for multiple difference table. This method differs from existing methods in that:

• It is much faster
• It uses a unique pattern triangle.

## Graphical abstract

Graphical abstract

**Figure:**
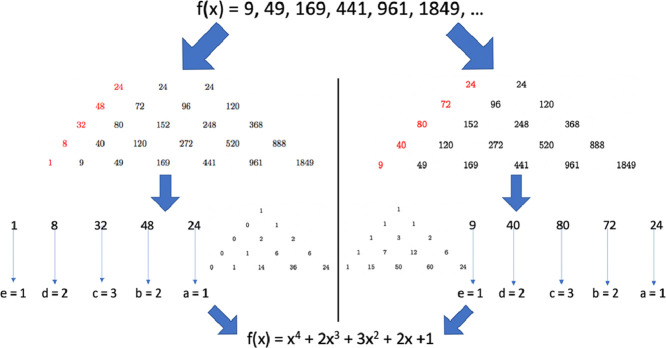

## Direct submission or co-submission

Co-submissions are papers that have been submitted alongside an original research paper accepted for publication by another Elsevier journal

## Specifications Table

**Table:** 

Subject Area	Computer Science
More specific subject area	Identifying a Function
Method name	Method: Split Difference Pattern Triangles: 1st Difference and Difference Zero Triangles
Name and reference of original method	System of Equations http://www.math.cmu.edu/~bkell/21110–2010s/formula.html Method of Differences. Brilliant.org. Retrieved 21:28, May 9, 2020, from https://brilliant.org/wiki/method-of-differences/ Newton's Interpolation Formula https://www.encyclopediaofmath.org/index.php/Newton_interpolation_formula
Resource availability	

## Method details

 

## Introduction

Often in algebra, we see sequences and need to find the polynomial that fits that sequence. The current method known involves setting up an equation similar to:

$$y=ax^{3}+bx^{2}+\text{cx}+d$$

and solving a system of equations. This method uses a pattern to determine the coefficients of the polynomial rather than the extensive method that we use today.

## Experimental and computational details

### Split difference

#### Finding the differences

##### Splitting the sequence into its differences

Given are the first few terms of the sequence:

9,49,169,441,961,1849

There is a function that can resemble this sequence. The first step would be to split the sequence into its differences. To do so, the differences of the terms would be found. Then, the differences of those differences are found, and this process can be repeated till all the differences are the same.

Example:

**Table:** 

Row 4					24		24				
Row 3				72		96		120			
Row 2			80		152		248		368		
Row 1		40		120		272		520		888	
Row 0	9		49		169		441		961		1849

##### Determine the degree of the polynomial

If the function is a polynomial of some whole number degree, the successive differences between the differences should at one point be the same. The number of rows of differences obtain would be the degree of the polynomial.

Example:

Row 4 shows constant differences. Therefore, the degree of the polynomial equation that represents the given sequence is 4.

##### Finding the 0th differences

For this method it is necessary to start at the 0th term and its successive differences. When given a sequence that starts at the first term, we can use its difference table to find the 0th differences. Once the triangle of differences is found, the 0th term can be found by working backwards and finding the 0th differences.

Example:

**Table:** 

Row 4					24		24		24				
Row 3				48		72		96		120			
Row 2			32		80		152		248		368		
Row 1		8		40		120		272		520		888	
Row 0	1		9		49		169		441		961		1849

Now, you should have the following differences for each row:

1,8,32,48,24

#### Creating the term zero difference triangle

Begin with the triangle:

$$\begin{array}{cc} \;1\\ \;0 & \;1 \end{array}$$

The next step is to add another row. This is done by adding the numbers above it, similar to the rule used in Pascal's Triangle. However, now we will multiply each sum by their own term number. The leftmost number of each row will have the term number of 0. This process must be repeated till there are one plus the degree found in part one number of rows. Like Pascal's Triangle, this pattern triangle is the same every time you use this method.

Repeating this pattern will give you the triangle:

**Table:** 

Row 0					1				
Row 1				0		1			
Row 2			0		2		2		
Row 3		0		1		6		6	
Row 4	0		1		14		36		24

#### Apply and solve

##### Finding the coefficients

The final step is to piece together parts 1 and 2 to determine the function.

To do so, the 0th differences found in part 1 is used. First, the final difference is divided by the last term of the last row of the triangle in part 2. That value will be the leading coefficient. The rest of the numbers of that row is multiplied by that coefficient and subtracted from the other differences respectively. While going up the triangle, this process will be repeated till all of the numbers have been used up.

Example:

Begin with the differences found in Part 1.

1,8,32,48,24

Divide the last term of the differences by the corresponding term in the pattern triangle and subtract. Repeat until all the numbers in the pattern triangle are used.

$$\begin{array}{ccc} & & 1\;\;8\;\;32\;\;48\\ & & \;\;\;\;\frac{24}{24}\\ & & \;\;\;\;=1\\ & & \frac{0\;\;1\;\;14\;\;36}{1\;\;7\;\;18\;\;\frac{12}{6}}\\ & & \;\;\;=2\\ & & \frac{0\;\;2\;\;12}{1\;\;5\;\;\frac{6}{2}}\\ & & \;\;=3\\ & & \frac{0\;\;3}{1\;\;\frac{2}{1}}\\ & & \;\;=2\\ & & \frac{0}{\frac{1}{1}}\\ & & \;=1 \end{array}$$

##### Determine the equation

Here, we use the empty polynomial equation of the degree you found in Part 1, as shown below:

$$y=ax^{4}+bx^{3}+cx^{2}+\text{dx}+e$$

Substitute the variables in the equation above with the quotients found earlier in Part 3 (a is the first quotient).

Example:

$$y=x^{4}+2x^{3}+3x^{2}+2x+1$$

## Alternate form of split difference

There is a similar method to split difference which can be found to be easier in some circumstances.

### Finding the differences

Repeat the Part 1 process from the standard Split Difference method. However, there is no need to find the 0th term or the 0th differences.

Example:

**Table:** 

Row 4					24		24				
Row 3				72		96		120			
Row 2			80		152		248		368		
Row 1		40		120		272		520		888	
Row 0	9		49		169		441		961		1849

### Creating the first term difference triangle

Begin with the triangle:

$$\begin{array}{c} 1\\ 1\;\;\;\;\;\;\;\;\;\;\;\;\;1 \end{array}$$

Create another row by adding the numbers above times the term number each number holds, starting with left as 1. For example, the second number in the next row would be (1 * 1) + (1 * 2). Once again, create as many rows as the degree plus 1. Like Pascal's Triangle, this pattern triangle is the same every time you use this method.

**Table:** 

Row 0					1				
Row 1				1		1			
Row 2			1		3		2		
Row 3		1		7		12		6	
Row 4	1		15		50		6		24

### Apply and solve - Here, the method works same as it did in part 1

#### Finding the coefficients

The final step is to piece together parts 1 and 2 to determine the function. To do so, the 0th differences found in part 1 is used. First, the final difference is divided by the last term of the last row of the triangle in part 2. That value will be the leading coefficient. The rest of the numbers of that row is multiplied by that coefficient and subtracted from the other differences respectively. While going up the triangle, this process will be repeated till all of the numbers have been used up.

Example:

Begin with the differences found in Part 1.

9,40,80,72,24

Divide the last term of the differences by the corresponding term in the pattern triangle and subtract. Repeat until all the numbers in the pattern triangle are used.

Example:

$$\begin{array}{ccc} & & 9\;\;40\;\;80\;\;72\\ & & \;\;\;\;\;\frac{24}{24}\\ & & \;\;\;\;\;=1\\ & & \frac{1\;\;15\;\;50\;\;60}{8\;\;25\;\;30\;\;\frac{12}{6}}\\ & & \;\;\;\;\;=2\\ & & \frac{2\;\;14\;\;24}{6\;\;11\;\;\frac{6}{2}}\\ & & \;\;\;=3\\ & & \frac{3\;\;9}{3\;\;\frac{2}{1}}\\ & & \;\;=2\\ & & \frac{2}{\frac{1}{1}}\\ & & \;=1 \end{array}$$

#### Determine the equation

Here, we use the empty polynomial equation of the degree you found in Part 1, as shown below:

$$y=ax^{4}+bx^{3}+cx^{2}+\text{dx}+e$$

Substitute the variables in the equation above with the quotients found earlier in Part 3

Example:

$$y=x^{4}+2x^{3}+3x^{2}+2x+1$$

## Current methods

### System of equations [1]

The current method works by using a system of equations to solve the polynomial. One must set up multiple equations following the structure:

$$y=ax^{4}+bx^{3}+cx^{2}+\text{dx}+e$$

They will then substitute x for 1 and y for the first term and so on till they have as many equations as coefficients. Using the elimination method, this method requires you to find the coefficients one by one. However, this method can become very tedious, especially when dealing with higher degree polynomials. It is also very difficult when dealing with non-integer coefficients.

### Difference table [2]

The Split Difference Method is certainly based off the ideas behind this method. This method requires creating the difference table for the sequence to find the leading coefficient and highest degree. Then, the method calls for subtracting this value from each term in the sequence and drawing the difference table once more to find the next highest degree. This process is repeated until all the terms are found. However, this method is extremely time consuming as it requires one to create multiple difference tables.

### Newton's interpolation method [3]

This method involves creating a special difference table where the ‘difference’ between each successive term is found using a special equation. While this method may be fast, it doesn't provide one with a simplified polynomial function, but rather a quite large one that will take too much time to simplify at higher degrees.

### Split difference method benefits

This method compared to any previous method runs much faster, especially at higher degree equations. The first step of finding the differences and degree is the same as you would in any other method because it is necessary to determine the degree of the function. The triangle is one that doesn't change based on the scenario and therefore can be memorized for quick use. The only extra calculation occurs in the final step which is comprised of simple arithmetic and very little of it. This minimizes the issue of making an accidental error which tends to be common in the system of equations method. By reusing the difference table that would need to have been made anyways and limiting the method to only one difference table, we get a much faster process than that of the System of Equations and the Difference Table method. Additionally, we get a simplified result unlike that of Newton's Interpolation Method.

## Proof of pattern triangle

### Proof of the first term difference triangle

$$f\left(x\right)=A_{d}x^{d}+A_{d-1}x^{d-1}+A_{d-2}x^{d-2}+\ldots +A_{0}x^{0}$$

**Table:** 

x	f(x)/Δ_0_	Δ_1_	Δ_2_	Δ_3_	Δ_4_	Δ_n_

1	f(1)					
		f(2) – f(1)				
2	f(2)		f(3) – 2f(2) + *f*(1)			
		f(3) – f(2)		f(4) – 3f(3) + 3f(2) – f(1)		
3	f(3)		f(4) – 2f(3) + *f*(2)		f(5) – 4f(4) + 6f(3) – 4f(2) + *f*(1)	
		f(4) – f(3)		f(5) – 3f(4) + 3f(3) – f(2)		$\sum_{k=0}^{n}{\left(-1\right)}^{n}\left(\begin{array}{c} n\\ k \end{array}\right)f\left(n-k+1\right)$
4	f(4)		f(5) – 2f(4) + *f*(3)		f(6) – 4f(5) + 6f(4) – 4f(3) + *f*(2)	
		f(5) – f(4)		f(6) – 3f(5) + 3f(4) – f(3)		
5	f(5)		f(6) – 2f(5) + *f*(4)			
		f(6) – f(5)				
6	f(6)					

Given the degree, d, is n when Δ*_n_* is constant, the leading coefficient, A_d_, is:

$$\frac{\sum_{k=0}^{n}{\left(-1\right)}^{n}\left(\begin{array}{c} n\\ k \end{array}\right)f\left(n-k+1\right)}{n!}$$

Let g(x) = *f*(x) – A_d_x^d^

**Table:** 

x	g(x) or Δ_0_	Δ_1_	Δ_2_	Δ_3_	Δ_n_

1	g(1)				
		g(2) – g(1)			
2	g(2)		g(3) – 2g(2) + *g*(1)		
		g(3) – g(2)		g(4) – 3g(3) + 3g(2) – g(1)	
3	g(3)		g(4) – 2g(3) + *g*(2)		$\sum_{k=0}^{n}{\left(-1\right)}^{n}\left(\begin{array}{c} n\\ k \end{array}\right)g\left(n-k+1\right)$
		g(4) – g(3)		g(5) – 3 g(4) + 3 g(3) – g(2)	
4	g(4)		g(5) – 2g(4) + *g*(3)		$\sum_{k=0}^{n}{\left(-1\right)}^{n}\left(\begin{array}{c} n\\ k \end{array}\right)g\left(n-k+2\right)$
		g(5) – g(4)		g(6) – 3g(5) + 3g(4) – g(3)	
5	g(5)		g(6) – 2g(5) + *g*(4)		
		g(6) – g(5)			
6	g(6)				

Substitute f(x) – A_d_x^d^ for g(x).

**Table:** 

x	g(x) or Δ_0_	Δ_1_	Δ_2_	Δ_3_	Δ_n_

1	f(1) – A_d_1^d^				
		(f(2) – A_d_2^d^) – (f(1) – A_d_1^d^)			
2	f(2) – A_d_2^d^		(f(3) – A_d_3^d^) – 2(f(2) – A_d_2^d^) + (f(1) – A_d_1^d^)		
		(f(3) – A_d_3^d^) – (f(2) – A_d_2^d^)		(f(4) – A_d_4^d^) – 3(f(3) – A_d_3^d^) + 3(f(2) – A_d_2^d^) – (f(1) – A_d_1^d^)	
3	f(3) – A_d_3^d^		(f(4) – A_d_4^d^) – 2(f(3) – A_d_3^d^) +(f(2) – A_d_2^d^)		$\sum_{k=0}^{n}{\left(-1\right)}^{n}\left(\begin{array}{c} n\\ k \end{array}\right)[f(n-k+1)-$$A_{d}{\left(n-k+1\right)}^{d}]$
		(f(4) – A_d_4^d^) – (f(3) – A_d_3^d^)		(f(5) – A_d_5^d^) – 3(f(4) – A_d_4^d^) +3(f(3) – A_d_3^d^) – (f(2) – A_d_2^d^)	
4	f(4) – A_d_4^d^		(f(5) – A_d_5^d^) – 2(f(4) – A_d_4^d^) +(f(3) – A_d_3^d^)		$\sum_{k=0}^{n}{\left(-1\right)}^{n}\left(\begin{array}{c} n\\ k \end{array}\right)[f(n-k+2)-$$A_{d}{\left(n-k+2\right)}^{d}]$
		(f(5) – A_d_5^d^) – (f(4) – A_d_4^d^)		(f(6) – A_d_6^d^) – 3(f(5) – A_d_5^d^) +3(f(4) – A_d_4^d^) – (f(3) – A_d_3^d^)	
5	f(5) – A_d_5^d^		(f(6) – A_d_6^d^) – 2(f(5) – A_d_5^d^) +(f(4) – A_d_4^d^)		
		(f(6) – A_d_6^d^) – (f(5) – A_d_5^d^)			
6	f(6) – A_d_6^d^				

Given the degree of g(x), d-1, is n when Δ*_n_* is constant, the leading coefficient, A_d-1_, is:

$$\frac{\sum_{k=0}^{n}{\left(-1\right)}^{n}\left(\begin{array}{c} n\\ k \end{array}\right)\left[f\left(n-k+1\right)-A_{d}{\left(n-k+1\right)}^{d}\right]}{n!}$$

Using the previous two coefficients, we can let h(x) = *f*(x) – A_d_x^d^ – A_d-1_x^d-1^ .

The first term of Δ_n_ for h(x) would be:

$$\sum_{k=0}^{n}{\left(-1\right)}^{n}\left(\begin{array}{c} n\\ k \end{array}\right)\left[f\left(n-k+1\right)-A_{d}{\left(n-k+1\right)}^{d}-\;A_{d-1}{\left(n-k+1\right)}^{d-1}\right]$$

If this process is repeated until every coefficient is found, the function will only have the leading coefficient. We can call this function z(x).

$$z\left(x\right)=f\left(x\right)-A_{d}x^{d}-A_{d-1}x^{d-1}-\cdots -A_{1}x^{1}=A_{0}x^{0}$$

The first term of Δ_n_ for z(x) would be:

$$\sum_{k=0}^{n}{\left(-1\right)}^{n}\left(\begin{array}{c} n\\ k \end{array}\right)\left[f\left(n-k+1\right)-A_{d}{\left(n-k+1\right)}^{d}-\;A_{d-1}{\left(n-k+1\right)}^{d-1}-\ldots -A_{1}{\left(n-k+1\right)}^{1}\right]$$

Since z(x) is a constant coefficient function, there is only one Δ_n_ to pay attention to - Δ_0_. All other columns will be 0.

Thus, we can simplify the summation above to:

$$\sum_{k=0}^{n}{\left(-1\right)}^{n}\left(\begin{array}{c} n\\ k \end{array}\right)\left[f\left(n-k+1\right)-\sum_{i=0}^{d}A_{d-i}{\left(n-k+1\right)}^{d-i}\right]$$

Now we can define a function, P_m_(x) which represents a part of the original polynomial, f(x) with degree d, where m represents the degree of the function. The general formula for first difference of each column Δ_n_ for P_m_(x) would be:

$$\sum_{k=0}^{n}{\left(-1\right)}^{n}\left(\begin{array}{c} n\\ k \end{array}\right)\left[f\left(n-k+1\right)-\sum_{i=0}^{d-m-1}A_{d-i}{\left(n-k+1\right)}^{d-i}\right]$$

Now if we write the first term of each column Δ_n_ for any degree, d we get:

**Table:** 

P_M_	0	1	2	N

0	$f\left(1\right)-\left[\sum_{i=0}^{d-1}A_{d-i}{\left(1\right)}^{d-i}\right]$			
1	$f\left(1\right)-\left[\sum_{i=0}^{d-2}A_{d-i}{\left(1\right)}^{d-i}\right]\sum_{k=0}^{1}{\left(-1\right)}^{k}\left[f\left(2-k\right)-\sum_{i=0}^{d-2}A_{d-i}{\left(2-k\right)}^{d-i}\right]$			
2	$f\left(1\right)-\left[\sum_{i=0}^{d-3}A_{d-i}{\left(1\right)}^{d-i}\right]$	$\sum_{k=0}^{1}{\left(-1\right)}^{k}[f(2-k)-$$\sum_{i=0}^{d-3}A_{d-i}{\left(2-k\right)}^{d-i}]$	$\sum_{k=0}^{2}{\left(-1\right)}^{k}\left(\begin{array}{c} n\\ k \end{array}\right)[f(3-k)-$$\sum_{i=0}^{d-3}A_{d-i}{\left(3-k\right)}^{d-i}]$	
…				
D–3	$f\left(1\right)-\left[\sum_{i=0}^{2}A_{d-i}{\left(1\right)}^{d-i}\right]$	$\sum_{k=0}^{1}{\left(-1\right)}^{k}[f(2-k)-$$\sum_{i=0}^{2}A_{d-i}{\left(2-k\right)}^{d-i}]$	$\sum_{k=0}^{2}{\left(-1\right)}^{k}\left(\begin{array}{c} n\\ k \end{array}\right)[f(3-k)-$$\sum_{i=0}^{2}A_{d-i}{\left(3-k\right)}^{d-i}]$	$\sum_{k=0}^{1}{\left(-1\right)}^{k}\left(\begin{array}{c} n\\ k \end{array}\right)[f(n-k+1)-$$\sum_{i=0}^{2}A_{d-i}{\left(n-k+1\right)}^{d-i}]$
D–2	$f\left(1\right)-\left[\sum_{i=0}^{1}A_{d-i}{\left(1\right)}^{d-i}\right]$	$\sum_{k=0}^{1}{\left(-1\right)}^{k}[f(2-k)-$$\sum_{i=0}^{1}A_{d-i}{\left(2-k\right)}^{d-i}]$	$\sum_{k=0}^{2}{\left(-1\right)}^{k}\left(\begin{array}{c} n\\ k \end{array}\right)[f(3-k)-$$\sum_{i=0}^{1}A_{d-i}{\left(3-k\right)}^{d-i}]$	$\sum_{k=0}^{1}{\left(-1\right)}^{k}\left(\begin{array}{c} n\\ k \end{array}\right)[f(n-k+1)-$$\sum_{i=0}^{1}A_{d-i}{\left(n-k+1\right)}^{d-i}]$
D–1	f(1) – A_d_1^d^	f(2) – f(1) – A_d_2^d^ + A_d_1^d^	$\sum_{k=0}^{2}{\left(-1\right)}^{k}\left(\begin{array}{c} n\\ k \end{array}\right)[f(3-k)-$$\sum_{i=0}^{0}A_{d-i}{\left(3-k\right)}^{d-i}]$	$\sum_{k=0}^{1}{\left(-1\right)}^{k}\left(\begin{array}{c} n\\ k \end{array}\right)[f(n-k+1)-$$\sum_{i=0}^{0}A_{d-i}{\left(n-k+1\right)}^{d-i}]$
D	f(1)	f(2) – f(1)	f(3) – 2f(2) + *f*(1)	$\sum_{k=0}^{n}{\left(-1\right)}^{k}\left(\begin{array}{c} n\\ k \end{array}\right)f\left(n-k+1\right)$

Taking the difference between the rows, without the last value in each row, we get:

**Table:** 

P_M_ – P_M-1_	0	1	2	N

P_0_ - 0				
P_1_ – P_0_	A_1_1^1^			
P_2_– P_1_	A_2_1^2^	A_2_(2^2^ – 1^2^)		
P_3_ – P_2_	A_3_1^3^	A_3_(2^3^ – 1^3^)	A_3_(3^3^ – 2(2)^3^ + 1^3^)	
…				
P_D-3_ – P_D-4_	$A_{d-3}\sum_{k=0}^{0}{\left(-1\right)}^{k}\left(\begin{array}{c} 0\\ k \end{array}\right){\left(1-k\right)}^{d-3}$	$A_{d-3}\sum_{k=0}^{1}{\left(-1\right)}^{k}\left(\begin{array}{c} 1\\ k \end{array}\right){\left(2-k\right)}^{d-3}$	$A_{d-3}\sum_{k=0}^{2}{\left(-1\right)}^{k}\left(\begin{array}{c} 2\\ k \end{array}\right){\left(3-k\right)}^{d-3}$	$A_{d-3}\sum_{k=0}^{n}{\left(-1\right)}^{k}\left(\begin{array}{c} n\\ k \end{array}\right){\left(n-k+1\right)}^{d-3}$
P_D-2_ – P_D-3_	$A_{d-2}\sum_{k=0}^{0}{\left(-1\right)}^{k}\left(\begin{array}{c} 0\\ k \end{array}\right){\left(1-k\right)}^{d-2}$	$A_{d-2}\sum_{k=0}^{1}{\left(-1\right)}^{k}\left(\begin{array}{c} 1\\ k \end{array}\right){\left(2-k\right)}^{d-2}$	$A_{d-2}\sum_{k=0}^{2}{\left(-1\right)}^{k}\left(\begin{array}{c} 2\\ k \end{array}\right){\left(3-k\right)}^{d-2}$	$A_{d-2}\sum_{k=0}^{n}{\left(-1\right)}^{k}\left(\begin{array}{c} n\\ k \end{array}\right){\left(n-k+1\right)}^{d-2}$
P_D-1_ – P_D-2_	$A_{d-1}\sum_{k=0}^{0}{\left(-1\right)}^{k}\left(\begin{array}{c} 0\\ k \end{array}\right){\left(1-k\right)}^{d-1}$	$A_{d-1}\sum_{k=0}^{1}{\left(-1\right)}^{k}\left(\begin{array}{c} 1\\ k \end{array}\right){\left(2-k\right)}^{d-1}$	$A_{d-1}\sum_{k=0}^{2}{\left(-1\right)}^{k}\left(\begin{array}{c} 2\\ k \end{array}\right){\left(3-k\right)}^{d-1}$	$A_{d-1}\sum_{k=0}^{n}{\left(-1\right)}^{k}\left(\begin{array}{c} n\\ k \end{array}\right){\left(n-k+1\right)}^{d-1}$
P_D_ – P_D-1_	$A_{d}\sum_{k=0}^{0}{\left(-1\right)}^{k}\left(\begin{array}{c} 0\\ k \end{array}\right){\left(1-k\right)}^{d}$	$A_{d}\sum_{k=0}^{1}{\left(-1\right)}^{k}\left(\begin{array}{c} 1\\ k \end{array}\right){\left(2-k\right)}^{d}$	$A_{d}\sum_{k=0}^{2}{\left(-1\right)}^{k}\left(\begin{array}{c} 2\\ k \end{array}\right){\left(3-k\right)}^{d}$	$A_{d}\sum_{k=0}^{n}{\left(-1\right)}^{k}\left(\begin{array}{c} n\\ k \end{array}\right){\left(n-k+1\right)}^{d}$

With the last value in each row being:

$$\sum_{k=0}^{n}{\left(-1\right)}^{n}\left(\begin{array}{c} n\\ k \end{array}\right)\left[f\left(n-k+1\right)-\sum_{i=0}^{d-m-1}A_{d-i}{\left(n-k+1\right)}^{d-i}\right]\;=m!\;$$

This pattern is similar to the pattern triangle which shows the values as:

$$\begin{array}{c} 1\\ 1\;\;\;\;\;\;1\\ 1\;\;\;\;\;\;3\;\;\;\;\;\;\;2\\ 1\;\;\;\;\;\;7\;\;\;\;\;\;12\;\;\;\;\;\;6\\ 1\;\;\;\;\;15\;\;\;\;\;50\;\;\;\;\;60\;\;\;\;24 \end{array}$$

The difference between this pattern triangle and the table we have above is that the constants behind each number in the pattern triangle are set as 1.

Simplify the following equation

$$\left(n+1\right)\left[\sum_{k=0}^{n}{\left(-1\right)}^{k}\left(\begin{array}{c} n\\ k \end{array}\right){\left(n-k+1\right)}^{m}\right]+\left(n+2\right)\left[\sum_{k=0}^{n+1}{\left(-1\right)}^{k}\left(\begin{array}{c} n+1\\ k \end{array}\right){\left(n-k+2\right)}^{m}\right]$$

Expand the summation:

$$\begin{array}{ccc} & = & \left(n+1\right)\left[\left(\begin{array}{c} n\\ 0 \end{array}\right){\left(n+1\right)}^{m}-\left(\begin{array}{c} n\\ 1 \end{array}\right){\left(n\right)}^{m}+\left(\begin{array}{c} n\\ 2 \end{array}\right){\left(n-1\right)}^{m}-\cdots +{\left(-1\right)}^{n}\left(\begin{array}{c} n\\ n \end{array}\right){\left(1\right)}^{m}\right]\\ & & +\left(n+2\right)\left[\left(\begin{array}{c} n+1\\ 0 \end{array}\right){\left(n+2\right)}^{m}-\left(\begin{array}{c} n+1\\ 1 \end{array}\right){\left(n+1\right)}^{m}+\left(\begin{array}{c} n+1\\ 2 \end{array}\right){\left(n\right)}^{m}-\cdots +{\left(-1\right)}^{n+1}\left(\begin{array}{c} n+1\\ n+1 \end{array}\right){\left(1\right)}^{m}\right] \end{array}$$

Distribute:

$$\begin{array}{ccc} & = & s\left(n+1\right)\left(\begin{array}{c} n\\ 0 \end{array}\right){\left(n+1\right)}^{m}-\left(n+1\right)\left(\begin{array}{c} n\\ 1 \end{array}\right){\left(n\right)}^{m}+\left(n+1\right)\left(\begin{array}{c} n\\ 2 \end{array}\right){\left(n-1\right)}^{m}-\cdots +\left(n+1\right){\left(-1\right)}^{n}\left(\begin{array}{c} n\\ n \end{array}\right){\left(1\right)}^{m}\\ & & +\left(n+2\right)\left(\begin{array}{c} n+1\\ 0 \end{array}\right){\left(n+2\right)}^{m}-\left(n+2\right)\left(\begin{array}{c} n+1\\ 1 \end{array}\right){\left(n+1\right)}^{m}+\left(n+2\right)\left(\begin{array}{c} n+1\\ 2 \end{array}\right){\left(n\right)}^{m}-\cdots \\ & & +\left(n+2\right){\left(-1\right)}^{n+1}\left(\begin{array}{c} n+1\\ n+1 \end{array}\right){\left(1\right)}^{m} \end{array}$$

Group like terms:

$$\begin{array}{ccc} & = & \left(n+2\right)\left(\begin{array}{c} n+1\\ 0 \end{array}\right){\left(n+2\right)}^{m}+{\left(n+1\right)}^{m}\left[\left(n+1\right)\left(\begin{array}{c} n\\ 0 \end{array}\right)-\left(n+2\right)\left(\begin{array}{c} n+1\\ 1 \end{array}\right)\right]\\ & & -{\left(n\right)}^{m}\left[\left(n+1\right)\left(\begin{array}{c} n\\ 1 \end{array}\right)-\left(n+2\right)\left(\begin{array}{c} n+1\\ 2 \end{array}\right)\right]+{\left(n-1\right)}^{m}\left[\left(n+1\right)\left(\begin{array}{c} n\\ 2 \end{array}\right)-\left(n+2\right)\left(\begin{array}{c} n+1\\ 3 \end{array}\right)\right]-\cdots \\ & & +{\left(-1\right)}^{n}1^{m}\left[\left(n+1\right)\left(\begin{array}{c} n\\ n \end{array}\right)-\left(n+2\right)\left(\begin{array}{c} n+1\\ n+1 \end{array}\right)\right] \end{array}$$

Expand the combinations:

$$\begin{array}{ccc} & = & \left(n+2\right)\left(\begin{array}{c} n+1\\ 0 \end{array}\right){\left(n+2\right)}^{m}+{\left(n+1\right)}^{m}\left[\left(n+1\right)\frac{n!}{0!n!}-\left(n+2\right)\frac{\left(n+1\right)!}{1!n!}\right]\\ & & -{\left(n\right)}^{m}\left[\left(n+1\right)\frac{n!}{1!\left(n-1\right)!}-\left(n+2\right)\frac{\left(n+1\right)!}{2!\left(n-1\right)!}\right]\\ & & +{\left(n-1\right)}^{m}\left[\left(n+1\right)\frac{n!}{2!\left(n-2\right)!}-\left(n+2\right)\frac{\left(n+1\right)!}{3!\left(n-2\right)!}\right]-\cdots \\ & & +{\left(-1\right)}^{n}1^{m}\left[\left(n+1\right)\frac{n!}{n!0!}-\left(n+2\right)\frac{\left(n+1\right)!}{\left(n+1\right)!0!}\right] \end{array}$$

Factor out like terms from each group:

$$\begin{array}{ccc} & = & \left(n+2\right)\left(\begin{array}{c} n+1\\ 0 \end{array}\right){\left(n+2\right)}^{m}+{\left(n+1\right)}^{m+1}\left[\frac{\left(n+1\right)!}{0!\left(n+1\right)!}-\frac{\left(n+2\right)!}{1!\left(n+1\right)!}\right]\\ & & -{\left(n\right)}^{m+1}\left[\frac{\left(n+1\right)!}{1!\left(n\right)!}-\frac{\left(n+2\right)!}{2!\left(n\right)!}\right]+{\left(n-1\right)}^{m+1}\left[\frac{\left(n+1\right)!}{2!\left(n-1\right)!}-\frac{\left(n+2\right)!}{3!\left(n-1\right)!}\right]-\cdots \\ & & +{\left(-1\right)}^{n}1^{m}\left[\frac{\left(n+1\right)!}{\left(n+1\right)!0!}-\frac{\left(n+2\right)!}{\left(n+1\right)!0!}\right] \end{array}$$

Simplify into a summation:

$$\left(n+2\right)\left(\begin{array}{c} n+1\\ 0 \end{array}\right){\left(n+2\right)}^{m}+\sum_{k=0}^{n}{\left(-1\right)}^{k}{\left(n-k+1\right)}^{m+1}\left[\left(\begin{array}{c} n+1\\ k \end{array}\right)-\left(\begin{array}{c} n+2\\ k+1 \end{array}\right)\right]$$

Simply the inner combinations:

$$\begin{array}{ccc} \left(\begin{array}{c} n+1\\ k \end{array}\right)\text{-}\left(\begin{array}{c} n+2\\ k+1 \end{array}\right) & & =\frac{\left(n+1\right)!}{k!\left(n-k+1\right)!}-\frac{\left(n+2\right)!}{\left(k+1\right)!\left(n-k+1\right)!}\\ & & =\frac{\left(k+1\right)\left(n+1\right)!}{\left(k+1\right)!\left(n-k+1\right)!}-\frac{\left(n+2\right)!}{\left(k+1\right)!\left(n-k+1\right)!}\\ & & =\left(n+1\right)!\left[\frac{k+1\text{-}n\text{-}2}{\left(k+1\right)!\left(n-k+1\right)!}\right]=-\left[\frac{\left(n+1\right)!}{\left(k+1\right)!\left(n-k\right)!}\right]=-\left(\begin{array}{c} n+1\\ k+1 \end{array}\right) \end{array}$$

The expression above can be simplified to:

$$\begin{array}{ccc} & & =\left[\sum_{k=0}^{n+1}{\left(-1\right)}^{k}\left(\begin{array}{c} n+1\\ k \end{array}\right){\left(n-k+2\right)}^{m+1}\right]=\left(n+2\right)\left(\begin{array}{c} n+1\\ 0 \end{array}\right){\left(n+2\right)}^{m}+\sum_{k=0}^{n}{\left(-1\right)}^{k+1}\left(\begin{array}{c} n+1\\ k+1 \end{array}\right){\left(n-k+1\right)}^{m+1}\\ & & =\sum_{k=\text{-}1}^{n}{\left(-1\right)}^{k+1}\left(\begin{array}{c} n+1\\ k+1 \end{array}\right){\left(n-k+1\right)}^{m+1}=\sum_{k=0}^{n+1}{\left(-1\right)}^{k}\left(\begin{array}{c} n+1\\ k \end{array}\right){\left(n-k+2\right)}^{m+1}\\ & & ∴\left(n+1\right)\left[\sum_{k=0}^{n}{\left(-1\right)}^{k}\left(\begin{array}{c} n\\ k \end{array}\right){\left(n-k+1\right)}^{m}\right]+\left(n+2\right)\left[\sum_{k=0}^{n+1}{\left(-1\right)}^{k}\left(\begin{array}{c} n+1\\ k \end{array}\right){\left(n-k+2\right)}^{m}\right] \end{array}$$

Therefore, this follows the pattern of the First Term Difference Triangle

### Term zero difference triangle

The first pattern triangle can be derived in a similar way except we start with the *x* = 0 term instead of *x* = 1 since the pattern triangle uses the 0th differences rather than the 1st. The difference table would for the sequence would be:

**Table:** 

x	f(x) or Δ_0_	Δ_1_	Δ_2_	Δ_3_	Δ_4_	Δ_n_

0	f(0)					
		f(1) – f(0)				
1	f(1)		f(2) – 2f(1) + *f*(0)			
		f(2) – f(1)		f(3) – 3f(2) + 3f(1) – f(0)		
2	f(2)		f(3) – 2f(2) + *f*(1)		f(4) – 4f(3) + 6f(2) – 4f(1) + *f*(0)	
		f(3) – f(2)		f(4) – 3f(3) + 3f(2) – f(1)		$\sum_{k=0}^{n}{\left(-1\right)}^{n}\left(\begin{array}{c} n\\ k \end{array}\right)f\left(n-k\right)$
3	f(3)		f(4) – 2f(3) + *f*(2)		f(5) – 4f(4) + 6f(3) – 4f(2) + *f*(1)	
		f(4) – f(3)		f(5) – 3f(4) + 3f(3) – f(2)		$\sum_{k=0}^{n}{\left(-1\right)}^{n}\left(\begin{array}{c} n\\ k \end{array}\right)f\left(n-k+1\right)$
4	f(4)		f(5) – 2f(4) + *f*(3)		f(6) – 4f(5) + 6f(4) – 4f(3) + *f*(2)	
		f(5) – f(4)		f(6) – 3f(5) + 3f(4) – f(3)		
5	f(5)		f(6) – 2f(5) + *f*(4)			
		f(6) – f(5)				
6	f(6)					

Now if we write the first term of each column Δ_n_ for any degree, d we get:

**Table:** 

P_M_	0	1	2	N

0	$f\left(0\right)\text{--}\left[\sum_{i=0}^{d-1}A_{d-i}{\left(0\right)}^{d-i}\right]$			
1	$f\left(0\right)\text{--}\left[\sum_{i=0}^{d-2}A_{d-i}{\left(0\right)}^{d-i}\right]$	$\sum_{k=0}^{1}{\left(-1\right)}^{k}[f(1-k)-$$\sum_{i=0}^{d-2}A_{d-i}{\left(1-k\right)}^{d-i}]$		
2	$f\left(0\right)\text{--}\left[\sum_{i=0}^{d-3}A_{d-i}{\left(0\right)}^{d-i}\right]$	$\sum_{k=0}^{1}{\left(-1\right)}^{k}[f(1-k)-$$\sum_{i=0}^{d-3}A_{d-i}{\left(1-k\right)}^{d-i}]$	$\sum_{k=0}^{2}{\left(-1\right)}^{k}\left(\begin{array}{c} n\\ k \end{array}\right)[f(2-k)-$$\sum_{i=0}^{d-3}A_{d-i}{\left(2-k\right)}^{d-i}]$	
…				
d–3	$f\left(0\right)\text{--}\left[\sum_{i=0}^{2}A_{d-i}{\left(0\right)}^{d-i}\right]$	$\sum_{k=0}^{1}{\left(-1\right)}^{k}[f(1-k)-$$\sum_{i=0}^{2}A_{d-i}{\left(1-k\right)}^{d-i}]$	$\sum_{k=0}^{2}{\left(-1\right)}^{k}\left(\begin{array}{c} n\\ k \end{array}\right)[f(2-k)-$$\sum_{i=0}^{2}A_{d-i}{\left(2-k\right)}^{d-i}]$	$\sum_{k=0}^{1}{\left(-1\right)}^{k}\left(\begin{array}{c} n\\ k \end{array}\right)[f(n-k)-$$\sum_{i=0}^{2}A_{d-i}{\left(n-k\right)}^{d-i}]$
d–2	$f\left(0\right)\text{--}\left[\sum_{i=0}^{1}A_{d-i}{\left(0\right)}^{d-i}\right]$	$\sum_{k=0}^{1}{\left(-1\right)}^{k}[f(1-k)-$$\sum_{i=0}^{1}A_{d-i}{\left(1-k\right)}^{d-i}]$	$\sum_{k=0}^{2}{\left(-1\right)}^{k}\left(\begin{array}{c} n\\ k \end{array}\right)[f(2-k)-$$\sum_{i=0}^{1}A_{d-i}{\left(2-k\right)}^{d-i}]$	$\sum_{k=0}^{1}{\left(-1\right)}^{k}\left(\begin{array}{c} n\\ k \end{array}\right)[f(n-k)-$$\sum_{i=0}^{1}A_{d-i}{\left(n-k\right)}^{d-i}]$
d–1	f(0) – A_d_0^d^	f(1) – f(0) – A_d_1^d^ + A_d_0^d^	$\sum_{k=0}^{2}{\left(-1\right)}^{k}\left(\begin{array}{c} n\\ k \end{array}\right)[f(2-k)-$$\sum_{i=0}^{0}A_{d-i}{\left(2-k\right)}^{d-i}]$	$\sum_{k=0}^{1}{\left(-1\right)}^{k}\left(\begin{array}{c} n\\ k \end{array}\right)[f(n-k)-$$\sum_{i=0}^{0}A_{d-i}{\left(n-k\right)}^{d-i}]$
d	f(0)	f(1) – f(0)	f(2) – 2f(1) + *f*(0)	$\sum_{k=0}^{n}{\left(-1\right)}^{k}\left(\begin{array}{c} n\\ k \end{array}\right)f\left(n-k\right)$

The last difference is each row is still equal to that row factorial, which gives the degree of that partial polynomial. Taking the difference between the rows, without the last value in each row, we get:

**Table:** 

P_M_ – P_M-1_	0	1	2	N

P_0_ - 0				
P_1_– P_0_	A_1_0^1^			
P_2_ – P_1_	A_2_0^2^	A_2_(1^2^ – 0^2^)		
P_3_ – P_2_	A_3_0^3^	A_3_(1^3^ – 0^3^)	A_3_(2^3^ – 2(1)^3^+ 0^3^)	
…				
P_D-3_ – P_D-4_	$A_{d-3}\sum_{k=0}^{0}{\left(-1\right)}^{k}\left(\begin{array}{c} 0\\ k \end{array}\right){\left(-k\right)}^{d-3}$	$A_{d-3}\sum_{k=0}^{1}{\left(-1\right)}^{k}\left(\begin{array}{c} 1\\ k \end{array}\right){\left(1-k\right)}^{d-3}$	$A_{d-3}\sum_{k=0}^{2}{\left(-1\right)}^{k}\left(\begin{array}{c} 2\\ k \end{array}\right){\left(2-k\right)}^{d-3}$	$A_{d-3}\sum_{k=0}^{n}{\left(-1\right)}^{k}\left(\begin{array}{c} n\\ k \end{array}\right){\left(n-k\right)}^{d-3}$
P_D-2_ – P_D-3_	$A_{d-2}\sum_{k=0}^{0}{\left(-1\right)}^{k}\left(\begin{array}{c} 0\\ k \end{array}\right){\left(-k\right)}^{d-2}$	$A_{d-2}\sum_{k=0}^{1}{\left(-1\right)}^{k}\left(\begin{array}{c} 1\\ k \end{array}\right){\left(1-k\right)}^{d-2}$	$A_{d-2}\sum_{k=0}^{2}{\left(-1\right)}^{k}\left(\begin{array}{c} 2\\ k \end{array}\right){\left(2-k\right)}^{d-2}$	$A_{d-2}\sum_{k=0}^{n}{\left(-1\right)}^{k}\left(\begin{array}{c} n\\ k \end{array}\right){\left(n-k\right)}^{d-2}$
P_D-1_ – P_D-2_	$A_{d-1}\sum_{k=0}^{0}{\left(-1\right)}^{k}\left(\begin{array}{c} 0\\ k \end{array}\right){\left(-k\right)}^{d-1}$	$A_{d-1}\sum_{k=0}^{1}{\left(-1\right)}^{k}\left(\begin{array}{c} 1\\ k \end{array}\right){\left(1-k\right)}^{d-1}$	$A_{d-1}\sum_{k=0}^{2}{\left(-1\right)}^{k}\left(\begin{array}{c} 2\\ k \end{array}\right){\left(2-k\right)}^{d-1}$	$A_{d-1}\sum_{k=0}^{n}{\left(-1\right)}^{k}\left(\begin{array}{c} n\\ k \end{array}\right){\left(n-k\right)}^{d-1}$
P_D_ – P_D-1_	$A_{d}\sum_{k=0}^{0}{\left(-1\right)}^{k}\left(\begin{array}{c} 0\\ k \end{array}\right){\left(-k\right)}^{d}$	$A_{d}\sum_{k=0}^{1}{\left(-1\right)}^{k}\left(\begin{array}{c} 1\\ k \end{array}\right){\left(1-k\right)}^{d}$	$A_{d}\sum_{k=0}^{2}{\left(-1\right)}^{k}\left(\begin{array}{c} 2\\ k \end{array}\right){\left(2-k\right)}^{d}$	$A_{d}\sum_{k=0}^{n}{\left(-1\right)}^{k}\left(\begin{array}{c} n\\ k \end{array}\right){\left(n-k\right)}^{d}$

With the last value in each row being:

$$\sum_{k=0}^{n}{\left(-1\right)}^{n}\left(\begin{array}{c} n\\ k \end{array}\right)\left[f\left(n-k\right)-\sum_{i=0}^{d-m-1}A_{d-i}{\left(n-k\right)}^{d-i}\right]\;=\;m!\;\;\;$$

This pattern is similar to the pattern triangle which shows the values as:

$$\begin{array}{c} 1\\ 0\;\;\;\;\;\;1\\ 0\;\;\;\;\;\;1\;\;\;\;\;\;2\\ 0\;\;\;\;\;\;1\;\;\;\;\;\;6\;\;\;\;\;\;6\\ \;0\;\;\;\;\;\;1\;\;\;\;\;14\;\;\;\;\;36\;\;\;\;24\\ \;\;\;0\;\;\;\;\;\;1\;\;\;\;\;30\;\;\;\;150\;\;\;240\;\;\;120. \end{array}$$

The difference between this pattern triangle and the table we have above is that the constants behind each number in the pattern triangle are set as 1.

Simplify the following equation:

$$\left[n+1\right]\left[\sum_{k=0}^{n}{\left(-1\right)}^{k}\left(\begin{array}{c} n\\ k \end{array}\right){\left(n-k\right)}^{m}+\sum_{k=0}^{n+1}{\left(-1\right)}^{k}\left(\begin{array}{c} n+1\\ k \end{array}\right){\left(n-k+1\right)}^{m}\right]$$

Expand the summation:

$$\begin{array}{ccc} & = & \left(n+1\right)[\left(\begin{array}{c} n\\ 0 \end{array}\right){\left(n\right)}^{m}-\;\left(\begin{array}{c} n\\ 1 \end{array}\right){\left(n-1\right)}^{m}+\;\left(\begin{array}{c} n\\ 2 \end{array}\right){\left(n-2\right)}^{m}-\ldots +\;{\left(-1\right)}^{n}\left(\begin{array}{c} n\\ n \end{array}\right){\left(0\right)}^{m}+\left(\begin{array}{c} n+1\\ 0 \end{array}\right){\left(n+1\right)}^{m}\\ & & -\;\left(\begin{array}{c} n+1\\ 1 \end{array}\right){\left(n\right)}^{m}+\left(\begin{array}{c} n+1\\ 2 \end{array}\right){\left(n-1\right)}^{m}-\ldots +\;{\left(-1\right)}^{n+1}\left(\begin{array}{c} n+1\\ n+1 \end{array}\right){\left(0\right)}^{m}] \end{array}$$

Distribute:

$$\begin{array}{ccc} & = & \left(n+1\right)\left(\begin{array}{c} n\\ 0 \end{array}\right){\left(n\right)}^{m}-\left(n+1\right)\left(\begin{array}{c} n\\ 1 \end{array}\right){\left(n-1\right)}^{m}+\left(n+1\right)\left(\begin{array}{c} n\\ 2 \end{array}\right){\left(n\right)}^{m}-\cdots +\left(n+1\right){\left(-1\right)}^{n}\left(\begin{array}{c} n\\ n \end{array}\right){\left(0\right)}^{m}\\ & & +\left(n+1\right)\left(\begin{array}{c} n+1\\ 0 \end{array}\right){\left(n+1\right)}^{m}-\left(n+1\right)\left(\begin{array}{c} n+1\\ 1 \end{array}\right){\left(n\right)}^{m}+\left(n+1\right)\left(\begin{array}{c} n+1\\ 2 \end{array}\right){\left(n-1\right)}^{m}-\cdots \\ & & +\left(n+1\right){\left(-1\right)}^{n+1}\left(\begin{array}{c} n+1\\ n+1 \end{array}\right){\left(0\right)}^{m} \end{array}$$

Group like terms:

$$\begin{array}{ccc} & = & \left(\begin{array}{c} n+1\\ 0 \end{array}\right){\left(n+1\right)}^{m+1}+\left(n+1\right)[{\left(n\right)}^{m}\left[\left(\begin{array}{c} n\\ 0 \end{array}\right)-\left(\begin{array}{c} n+1\\ 1 \end{array}\right)\right]-{\left(n-1\right)}^{m}\left[\left(\begin{array}{c} n\\ 1 \end{array}\right)-\left(\begin{array}{c} n+1\\ 2 \end{array}\right)\right]\\ & & +{\left(n-2\right)}^{m}\left[\left(\begin{array}{c} n\\ 2 \end{array}\right)-\left(\begin{array}{c} n+1\\ 3 \end{array}\right)\right]-\cdots +{\left(-1\right)}^{n}0^{m}\left[\left(\begin{array}{c} n\\ n \end{array}\right)-\left(\begin{array}{c} n+1\\ n+1 \end{array}\right)\right]] \end{array}$$

Expand the combinations:

$$\begin{array}{ccc} & & \;=\;\left(n\;+\;2\right)\left(\begin{array}{c} n\;+\;1\\ 0 \end{array}\right){\left(n\;+\;2\right)}^{m}\;+\;{\left(n\;+\;1\right)}^{m}\left[\left(n\;+\;1\right)\frac{n!}{0!\;n!}-\left(n+2\right)\frac{\left(n+1\right)!}{1!\;n!}\right]\\ & & \;-{\left(n\right)}^{m}\left[\left(n+1\right)\frac{n!}{1!\left(n-1\right)!}-\left(n+2\right)\frac{\left(n+1\right)!}{2!\left(n-1\right)!}\right]\\ & & \;+{\left(n-1\right)}^{m}\left[\left(n+1\right)\frac{n!}{2!\left(n-2\right)!}-\left(n+2\right)\frac{\left(n+1\right)!}{3!\left(n-2\right)!}\right]-\ldots \\ & & \;+{\left(-1\right)}^{n}1^{m}\left[\left(n+1\right)\frac{n!}{n!0!}-\left(n+2\right)\frac{\left(n+1\right)!}{\left(n+1\right)!0!}\right] \end{array}$$

Combine the difference of factorials.

$$\begin{array}{ccc} & = & \left(\begin{array}{c} n+1\\ 0 \end{array}\right){\left(n+1\right)}^{m}+\left(n+1\right)[{\left(n\right)}^{m}\left[\frac{\left(1-\left(n+1\right)\right)\left(n\right)!}{1!\left(n\right)!}\right]-{\left(n-1\right)}^{m}\left[\frac{\left(2-\left(n+1\right)\right)\left(n\right)!}{2!\left(n-1\right)!}\right]\\ & & +{\left(n-2\right)}^{m}\left[\frac{\left(3-\left(n+1\right)\right)\left(n\right)!}{3!\left(n-2\right)!}\right]-\;\ldots +{\left(-1\right)}^{n-1}1^{m}\left[\frac{\left(n-\left(n+1\right)\right)\left(n\right)!}{\left(n\right)!1!}\right]+0]\\ & = & \left(\begin{array}{c} n+1\\ 0 \end{array}\right){\left(n+1\right)}^{m}+\left(n+1\right)[{\left(n\right)}^{m}\left[\frac{-\left(n\right)\left(n\right)!}{1!\left(n\right)!}\right]-{\left(n-1\right)}^{m}\left[\frac{-\left(n-1\right)\left(n\right)!}{2!\left(n-1\right)!}\right]\\ & & +{\left(n-2\right)}^{m}\left[\frac{-\left(n-2\right)\left(n\right)!}{3!\left(n-2\right)!}\right]-\ldots +{\left(-1\right)}^{n-1}1^{m}\left[\frac{-1\left(n\right)!}{\left(n\right)!1!}\right]+0] \end{array}$$

Factor out like terms.

$$\begin{array}{ccc} & = & \left(\begin{array}{c} n+1\\ 0 \end{array}\right){\left(n+1\right)}^{m}+\left(n+1\right)[{\left(n\right)}^{m+1}\left[\frac{-\left(n\right)!}{1!\left(n\right)!}\right]-{\left(n-1\right)}^{m+1}\left[\frac{-\left(n\right)!}{2!\left(n-2\right)!}\right]\\ & & +\;{\left(n-2\right)}^{m+1}\left[\frac{-\left(n\right)!}{3!\left(n-3\right)!}\right]-\ldots +{\left(-1\right)}^{n-1}1^{m+1}\left[\frac{-1\left(n\right)!}{\left(n\right)!1!}\right]+0] \end{array}$$

Distribute.

$$\begin{array}{ccc} & = & \left(\begin{array}{c} n+1\\ 0 \end{array}\right){\left(n+1\right)}^{m+1}+[{\left(n\right)}^{m+1}\left[\frac{-\left(n+1\right)!}{1!\left(n\right)!}\right]-{\left(n-1\right)}^{m+1}\left[\frac{-\left(n+1\right)!}{2!\left(n-2\right)!}\right]\\ & & +{\left(n-2\right)}^{m+1}\left[\frac{-\left(n+1\right)!}{3!\left(n-3\right)!}\right]-\ldots +{\left(-1\right)}^{n-1}1^{m+1}\left[\frac{-1\left(n+1\right)!}{\left(n\right)!1!}\right]+0] \end{array}$$

Simplify into a summation.

$$\begin{array}{c} =\left(\begin{array}{c} n+1\\ 0 \end{array}\right){\left(n+1\right)}^{m}+\sum_{k=1}^{n+1}{\left(-1\right)}^{k+1}\left(\begin{array}{c} n+1\\ k \end{array}\right){\left(n-k+1\right)}^{m+1}=\;\sum_{k=0}^{n}{\left(-1\right)}^{k+1}\left(\begin{array}{c} n+1\\ k \end{array}\right){\left(n-k+1\right)}^{m+1}\\ ∴\left[n+1\right]\left[\sum_{k=0}^{n}{\left(-1\right)}^{k}\left(\begin{array}{c} n\\ k \end{array}\right){\left(n-k\right)}^{m}+\sum_{k=0}^{n+1}{\left(-1\right)}^{k}\left(\begin{array}{c} n+1\\ k \end{array}\right){\left(n-k+1\right)}^{m}\right]=\sum_{k=0}^{n+1}{\left(-1\right)}^{k}\left(\begin{array}{c} n+1\\ k \end{array}\right){\left(n-k+1\right)}^{m+1} \end{array}$$

This follows the pattern of the Term Zero Difference Triangle

### Proof of application of pattern triangle

From the previous proof, we know that the first term of each column Δ_n_ for any degree is:

$$\sum_{k=0}^{n}{\left(-1\right)}^{n}\left(\begin{array}{c} n\\ k \end{array}\right)\left[f\left(n-k+1\right)-\sum_{i=0}^{d-m-1}A_{d-i}{\left(n-k+1\right)}^{d-i}\right]$$

We know that

$$\Delta_{d,n}-\Delta_{d-1,n}=A_{d}\sum_{k=0}^{n}{\left(-1\right)}^{k}\left(\begin{array}{c} n\\ k \end{array}\right){\left(n-k+1\right)}^{d}$$

where the summation gives each term in the pattern triangle. Thus, the following equation:

$$\Delta_{d-1,n}=\Delta_{d,n}-A_{d}\sum_{k=0}^{n}{\left(-1\right)}^{k}\left(\begin{array}{c} n\\ k \end{array}\right){\left(n-k+1\right)}^{d}$$

will give you the differences of the polynomial function one degree lower (removing the highest degree variable like shown in the proof of the pattern triangle for g(x)). Since A_d_ is the last term of each row divided by n!, the equation above can be simplified to:

$$\Delta_{d-1,n}=\Delta_{d,n}-\left[\frac{\sum_{k=0}^{n}{\left(-1\right)}^{n}\left(\begin{array}{c} n\\ k \end{array}\right)f\left(n-k+1\right)}{n!}\right]\sum_{k=0}^{n}{\left(-1\right)}^{k}\left(\begin{array}{c} n\\ k \end{array}\right){\left(n-k+1\right)}^{d}$$

This equation above means that the first term of each column in the difference table of the function P_d-1_(x) can be found by taking the first term of each column in the difference table of the function P_d_(x) and subtracting the quantity of each respective value in the pattern triangle times the value of the leading coefficient of P_d_(x), which can also be found by knowing the differences of P_d_(x).

It is important to understand that there can be an infinite amount number of polynomial equations that can represent the function. For example, if you were given the first two terms of a sequence – 0,1 – any equation of form f(x) = (x-1)^d^ will match the function, where d is all real numbers.

Therefore, if given the first n terms of a sequence, one can determine the lowest degree polynomial function of maximum degree, n – 1, that represents the sequence.

## Conclusion

In summary, the “Split Difference” method can be used to determine the equation to fit a given sequence. This method uses a pattern triangle and applies it to the difference table used to find the degree of a polynomial in order to speed up this process of determining a representative polynomial function. The experimental results have shown this method to be fast and very accurate.

A HEADINGS IN APPENDICES
A.1 INTRODUCTION
A.2 EXPERIMENTAL AND COMPUTATIONAL DETAILS
A.2.1 SPLIT DIFFERENCE
2.1.1 Finding the Differences
*2.1.1.1 Splitting the Sequence into its Differences*
*2.1.1.2 Determine the Degree of the Polynomial*
*2.1.1.3 Finding the 0th Differences*
2.1.2 Creating the Difference Zero Triangle
2.1.3 Apply and Solve
*2.1.3.1 Finding the Coefficients*
*2.1.3.2 Determine the Equation*
A.2.2 ALTERNATIVE METHOD
2.2.1 Finding the Differences
2.2.2 Creating the First Difference Triangle
2.2.3 Apply and Solve
*2.2.3.1 Finding the Coefficients*
*2.2.3.2 Determine the Equation*
A.3 CURRENT METHODS
A.3.1 System of Equations
A.3.2 Difference Table
A.3.3 Newton's Interpolation
A.3.4 Split Difference Method Benefits
A.4 PROOF
A.4.1 Proof of First Difference Pattern Triangle
A.4.2 Proof of Application of Pattern Triangle
A.4.3 Proof of Split Difference Methods
A.5 CONCLUSIONS

## Declaration of Competing Interests

The authors declare that they have no known competing financial interests or personal relationships that could have appeared to influence the work reported in this paper.
